# Endothelin-1 Plasma and Aqueous Humor Levels in Different Types of Glaucoma: A Systematic Review and Meta-Analysis

**DOI:** 10.3390/medicina60071117

**Published:** 2024-07-10

**Authors:** Stamatios Lampsas, Stylianos A. Kandarakis, Dionysios G. Vakalopoulos, Aikaterini Lampsa, Evangelos Oikonomou, Gerasimos Siasos, George D. Kymionis

**Affiliations:** 1 Second Department of Ophthalmology, Attikon University Hospital, Medical School, National and Kapodistrian University of Athens, 11528 Athens, Greece; 2 First Department of Ophthalmology, “G. Gennimatas” Hospital, Medical School, National and Kapodistrian University of Athens, 11528 Athens, Greece; 3 Third Department of Cardiology, Thoracic Diseases General Hospital Sotiria, Medical School, National and Kapodistrian University of Athens, 11528 Athens, Greece; 4 Cardiovascular Division, Harvard Medical School, Brigham and Women’s Hospital, Boston, MA 02115, USA

**Keywords:** endothelin-1, glaucoma, biomarker, vascular dysregulation, aqueous humor

## Abstract

*Background and Objectives*: Several studies suggest the complex relationship between Endothelin-1 (ET-1) levels with various types of glaucoma. This systematic review and meta-analysis explore ET-1 levels in plasma and aqueous humor among different types of glaucoma. *Materials and Methods*: A literature search (PubMed, ScienceDirect, Cochrane Library) was made up to April 2024 (PROSPERO: CRD42023430471). The results were synthesized according to PRISMA Guidelines. Results were presented as standardized mean differences (SMD) with 95% confidence intervals (CI). *Results*: A total of 2597 subjects (1513 patients with glaucoma vs. 1084 healthy controls) from 23 studies were included in a meta-analysis. Notably, patients with glaucoma reported significantly higher plasma levels of ET-1 compared to controls (SMD: 1.21, 95% CI: 0.59–1.82, *p* < 0.001). Particularly, plasma ET-1 levels were higher in primary open-angle glaucoma (POAG) (SMD: 0.87, 95% CI: 0.09–1.65, *p* < 0.05), normal-tension glaucoma (SMD: 0.86, 95% CI: 0.27–1.46, *p* = 0.05), and angle-closure glaucoma patients (SMD: 1.03, 95% CI: 0.43–1.63, *p* < 0.001) compared to healthy controls. Moreover, ET-1 aqueous humor levels were significantly higher in patients with glaucoma compared to controls (SMD: 1.60, 95% CI: 1.04–2.15, *p* < 0.001). In particular, aqueous humor levels were higher in POAG patients (SMD: 2.03 95% CI: 1.00–3.14, *p* < 0.001), and pseudoexfoliative glaucoma patients (SMD: 2.03, 95% CI: 1.00–3.07, *p* < 0.001) compared to controls. *Conclusions*: This meta-analysis indicates that elevated levels of ET-1 plasma and aqueous humor are significantly associated with different types of glaucoma. The pathogenesis of ET-1-related mechanisms may vary across different glaucoma types, indicating that possible therapeutic approaches targeting ET-1 pathways should be tailored to each specific glaucoma type.

## 1. Introduction

Glaucoma is a complex disease of ocular disorders causing a characteristic pattern of loss of retinal ganglion cells and damage to their axons, which damage the optic nerve head [1]. The global prevalence of glaucoma in the population aged 40 to 80 years is estimated at 3.54%, with a total of 76.0 million in 2020 and 111.8 million in 2040 people worldwide [2]. Several risk factors are implicated in glaucoma, such as elevated intraocular pressure (IOP), older age (>60 years old), thinner central cornea thickness, exfoliation syndrome, diabetes, and high blood pressure [3]. However, vascular dysregulation has come to light as a critical pathophysiological mechanism involved in glaucoma development and progression [4].

Considerable interest has emerged in Endothelin-1 (ET-1), a potent key modulator of ocular blood flow [5]. ET-1 has been characterized as a powerful vasoconstrictor, as it contributes to the basal constrictive tone of systemic vessels and may contribute to autoregulation of ocular blood flow and ocular vessel tone, especially in the choroid and optic nerve head [6,7]. ET-1 is a 21 amino acid peptide made by vascular endothelium throughout the body, including tissues relevant to glaucoma (non-pigmented ciliary epithelium, ciliary body muscle, and iris) [8]. Several studies have investigated the role of plasma and aqueous humor ET-1 levels, with most of them finding ET-1 plasma concentrations significantly elevated in glaucoma patients, and aqueous humor ET-1 levels to show great heterogeneity between studies and groups [9,10,11]. Findings from animal studies are also noteworthy as intravitreal injection of ET-1 in rats resulted in a dose-dependent and sustained reduction of optic nerve head blood flow [12], and ΕΤ-1 induced contraction in human trabecular meshwork cell cultures [13]. 

Although research interest is increasing to shed light on the pathophysiological mechanisms involved in ET-1 levels with the risk of glaucoma disease, the detailed picture of this connection is still obscure, especially among various types of glaucoma. This systematic review and meta-analysis summarizes the existing evidence on the role of ET-1 levels in both plasma and aqueous humor among glaucoma patients compared to healthy controls. In parallel, it also focuses individually on patients with each type of glaucoma, providing an up-to-date, comprehensive view of this relationship.

## 2. Materials and Methods

### 2.1. Literature Search

This study was designed according to the Preferred Reporting Items for Systematic reviews and Meta-Analysis (PRISMA) statement [14]. The protocol has been prospectively registered in PROSPERO (ID: CRD42023430471). PubMed, Cochrane Library, and ScienceDirect databases were searched up to 4 April 2024 for articles examining the role of Endothelin-1 plasma and aqueous humor levels on different types of glaucoma. The following search terms were used with an indicative search query to implement these criteria: “Endothelin-1” or “ET-1”. The keywords listed below were used to identify the outcome variables of interest: “Glaucoma”, “Primary open-angle Glaucoma”, “POAG”, “Angle-Closure Glaucoma”, “ACG”, “Normal Tension Glaucoma”, “NTG”, “Pseudoexfoliative Glaucoma”, or “PEXG”. Based on predefined selection criteria, two researchers independently evaluated the articles for eligibility. Any discrepancies were resolved through repeated reviewing and consensus among the authors. Database searches were rerun before final analyses and any appropriate further studies were retrieved for inclusion.

### 2.2. Study Selection and Data Extraction

All original observational studies (cohort, cross-sectional, and case-control) were included based on the following criteria for the included study design: (i) all studies were conducted in human adults (>18 years old); (ii) studies included soluble plasma and/or aqueous humor concentrations of ET-1 in patients with any type of glaucoma and healthy controls; (iii) studies included information about the diagnostic methods and the method of laboratory analysis of ET-1. We excluded from the analysis: (i) all reviews, systematic reviews, and meta-analyses; (ii) non-English language studies; (iii) animal studies; (iv) studies where the levels of ET-1 were unavailable (even after contacting the corresponding author). After the completion of screening, the following information was extracted from the included studies: (i) first author’s name; (ii) year of publication and title; (iii) study type/design; (iv) sample size (overall and per group); (v) study location (country); (vi) age and sex for the whole sample and each subgroup included. 

### 2.3. Quality Assessment and Publication Bias

The quality assessment and risk of bias assessment for the 23 studies meeting the eligibility criteria were conducted by the Newcastle–Ottawa Quality Assessment Scale (NOS) criteria, adapted for case-control, cross-sectional, and cohort studies respectively [15]. NOS assessment is based on three main domains: (i) The selection of participants, (ii) the comparability of the groups, and (iii) the ascertainment of either the exposure or outcome of interest. The maximum score is 9 for case-control and 10 for cross-sectional studies. We assigned scores of 0–3, 4–6, and 7–9 (or 7–10 for cross-sectional design) for low, moderate, and high quality, respectively. Also, publication bias was examined with funnel plot assessment, to evaluate the asymmetry of the values between the two groups under comparison. Outlier studies were removed using Egger’s test which is a statistical tool for quantifying funnel plot asymmetry. Study quality was not used as a criterion for outlier removal.

### 2.4. Statistical Analysis

The acquisition of data was achieved through either direct extraction or indirect calculation, following the methodology of Wan et al. formula in order to estimate mean and standard deviation (SD) in the case of data reported as median and interquartile range (IQR) [16]. To estimate differences, the standardized mean differences (SMD) of the compared groups and their 95% CIs were estimated using random effect models. The examination of statistical heterogeneity was carried out through Q statistic, based on the χ^2^ test (*p* = 0.05 as the significance threshold) and I^2^ was used to quantify the proportion of variance due to between-study heterogeneity. A sensitivity analysis was performed in the analysis of ET-1 levels (both in plasma and aqueous humor) when comparing SMDs between glaucoma patients and control subjects through a leave-one-out strategy. Moreover, a subgroup analysis was conducted between different types of glaucoma and control subjects when the included studies had at least two studies with the same comparison groups. A meta-analysis was conducted using RevMan 5.4 software (The Cochrane Collaboration, Oxford, UK).

## 3. Results

### 3.1. Search Results

**Figure 1:**
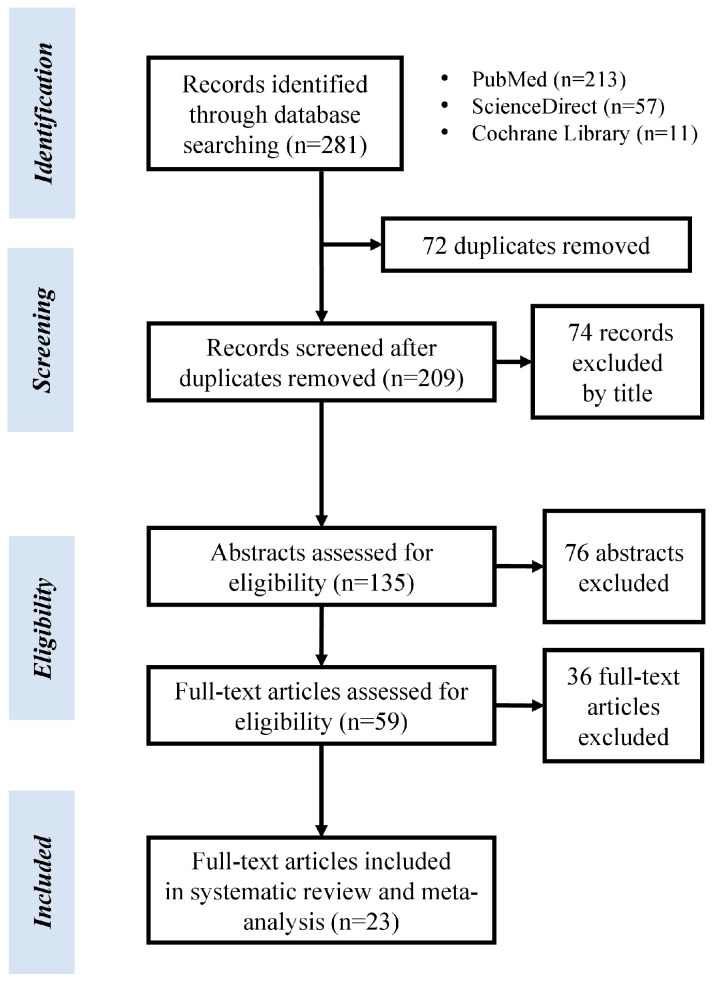
PRISMA flowchart for study selection.

A total of 281 studies was initially identified with 23 being eligible for inclusion in the systematic review and meta-analysis after the application of exclusion and inclusion criteria (Figure 1). Most of the studies included were case-control (*n* = 16, 69.6%), and the rest of them were cross-sectional studies (*n* = 7, 30.4%). There was a total sample size of *n* = 2597 subjects (1513 patients with glaucoma vs. 1084 healthy controls) (Table 1) [17,18,19,20,21,22,23,24,25,26,27,28,29,30,31,32,33,34,35,36,37,38,39]. 

### 3.2. Quality Assessment

The overall quality was found high for 19 studies (82.6%) and moderate for the remaining 4 (17.3%). Case-control studies displayed an average score of 7.3 out of 9, with 13 (81.3%) being of high quality. The same figure was 7.9 out of 10 for cross-sectional studies with 6 of them (85.7%) being of high quality (Supplementary Table S1). 

### 3.3. Quantitative Synthesis

#### 3.3.1. Plasma ET-1 Levels and Glaucoma

##### Plasma ET-1 Levels between Glaucoma Patients vs. Healthy Controls 

**Figure 2:**
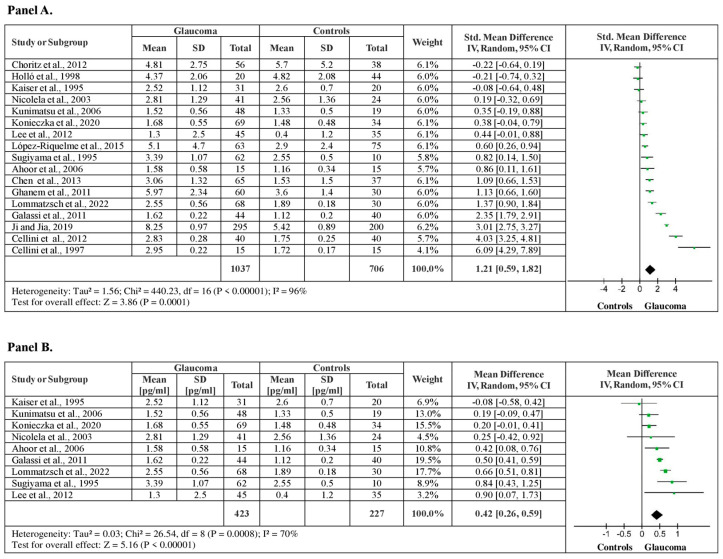
Plasma ET-1 levels between patients with glaucoma and healthy controls. (**A**) Forest plot displaying the meta-analysis of plasma ET-1 levels between glaucomatous patients and control subjects, their estimated pooled standardized mean difference (SMD), confidence interval (CI), and study heterogeneity; (**B**) Forest plot displaying the sensitivity analysis of plasma ET-1 levels between patients with glaucoma and control subjects, their estimated pooled mean difference (MD), confidence interval (CI), and study heterogeneity [17,18,19,20,21,22,23,24,26,27,29,31,32,33,34,35,39].

From the studies included in the meta-analysis, 17 studies examined plasma ET-1 levels between glaucoma patients and healthy controls. A total of 1743 subjects (1037 glaucoma patients vs. 706 healthy controls) were included in the analysis. The meta-analysis showed significantly higher plasma ET-1 levels for glaucoma patients, by a pooled SMD of 1.21 (95% CI: 0.59–1.82, *p* < 0.001) (Figure 2, Panel A). Although substantial heterogeneity (I^2^ = 96%, *p* < 0.001) was observed among studies, the sensitivity analysis confirmed the results. It showed higher ET-1 levels for glaucoma patients by a pooled mean difference of 0.42 pg/mL of ET-1 (95% CI: 0.26–0.59, *p* < 0.001), after funnel plot evaluation and 8 outlier studies removals, with moderate heterogeneity among studies (I^2^ = 70%, *p* < 0.001) (Figure 2, Panel B). 

##### Plasma ET-1 Levels between POAG Patients vs. Healthy Controls

**Figure 3:**
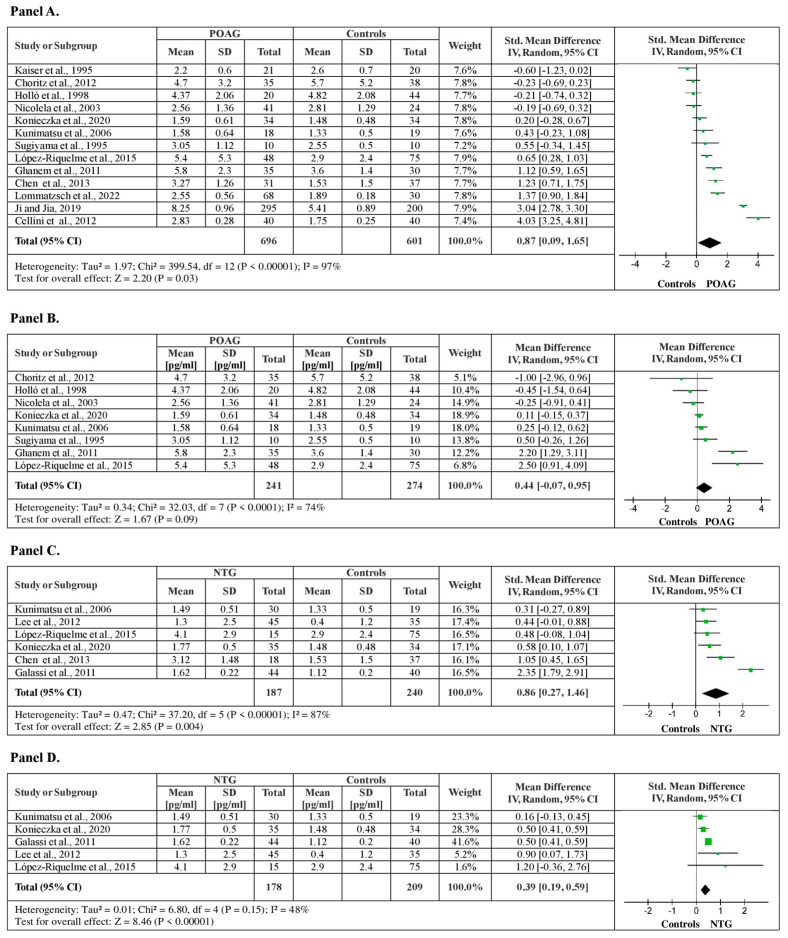
Plasma ET-1 levels between POAG, NTG patients, and healthy controls. (**A**) Forest plot displaying the meta-analysis of plasma ET-1 levels between POAG patients and control subjects, their estimated pooled standardized mean difference (SMD), confidence interval (CI), and study heterogeneity; (**B**) Forest plot displaying the sensitivity analysis of plasma ET-1 levels between patients with POAG and control subjects, their estimated pooled mean difference (MD), confidence interval (CI), and study heterogeneity; (**C**) Forest plot displaying the meta-analysis of plasma ET-1 levels between NTG patients and control subjects, their estimated pooled standardized mean difference (SMD), confidence interval (CI), and study heterogeneity; (**D**) Forest plot displaying the sensitivity analysis of plasma ET-1 levels between patients with NTG and control subjects, their estimated pooled mean difference (MD), confidence interval (CI), and study heterogeneity [19,20,21,22,23,24,26,27,29,31,32,33,34,35,39].

From the studies included in the meta-analysis, 13 studies examined plasma ET-1 levels between POAG patients and healthy controls, with a total of 1297 subjects (686 POAG patients vs. 601 healthy controls). The meta-analysis showed significantly higher plasma ET-1 levels in POAG patients, by a pooled SMD of 0.87 (95% CI: 0.09–1.65, *p* < 0.03), with a substantial heterogeneity among studies (I^2^ = 97%, *p* < 0.001) (Figure 3, Panel A). The sensitivity analysis confirmed the results showing higher ET-1 levels for POAG patients by a pooled mean difference of 0.44 pg/mL ET-1 (95% CI: −0.07–0.95, *p* < 0.001), after funnel plot evaluation and 5 outlier studies removals, with a moderate heterogeneity (I^2^ = 74%, *p* = 0.09) (Figure 3, Panel B).

##### Plasma ET-1 Levels between NTG Patients vs. Healthy Controls

In the present analysis, six studies investigating plasma ET-1 levels between NTG patients and healthy controls were included, with a total of 427 subjects (187 NTG patients vs. 601 healthy controls). The meta-analysis showed significantly higher plasma ET-1 levels in NTG patients, by a pooled SMD of 0.86 (95% CI: 0.27–1.46, *p* = 0.05), with a substantial heterogeneity among studies (I^2^ = 87%, *p* < 0.001) (Figure 3, Panel C). The sensitivity analysis confirmed the results showing higher ET-1 levels for NTG patients by a pooled MD of 0.39 pg/mL ET-1 (95% CI: 0.19–0.59, *p* < 0.001), after funnel plot evaluation and one outlier study removal, with a low heterogeneity among studies (I^2^ = 48%, *p* < 0.001) (Figure 3, Panel D).

##### Plasma ET-1 Levels between ACG Patients vs. Healthy Controls

**Figure 4:**
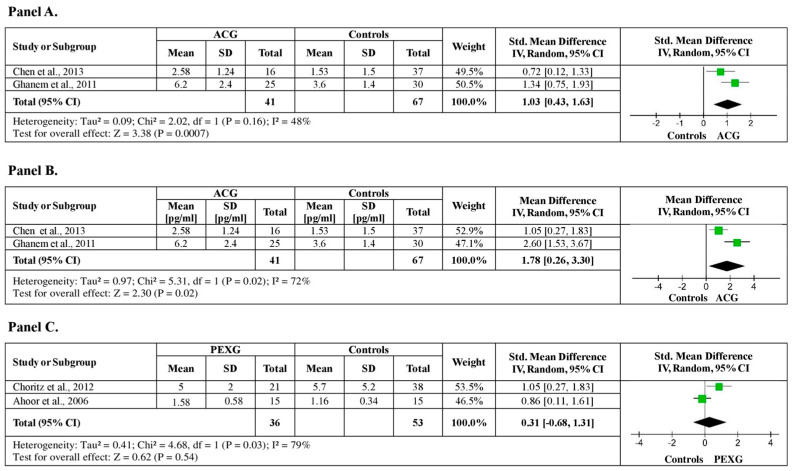
Plasma ET-1 levels between ACG, PEXG patients, and healthy controls. (**A**) Forest plot displaying the meta-analysis of plasma ET-1 levels between ACG patients and control subjects, their estimated pooled standardized mean difference (SMD), confidence interval (CI), and study heterogeneity; (**B**) Forest plot displaying the sensitivity analysis of plasma ET-1 levels between patients with ACG and control subjects, their estimated pooled mean difference (MD), confidence interval (CI), and study heterogeneity; (**C**) Forest plot displaying the meta-analysis of plasma ET-1 levels between PEXG patients and control subjects, their estimated pooled standardized mean difference (SMD), confidence interval (CI), and study heterogeneity [17,20,21,23].

Two studies were identified investigating plasma ET-1 levels between NTG patients and healthy controls, with a total of 108 subjects (41 ACG patients vs. 67 healthy controls). The analysis showed significantly higher plasma ET-1 levels, by a pooled SMD of 1.03 (95% CI: 0.43–1.63, *p* < 0.001) and low (I^2^ = 48%, *p* < 0.16) heterogeneity among studies (Figure 4, Panel A). Moreover, the analysis showed a pooled MD of 1.78 pg/mL ET-1 (95% CI: 0.26–3.30, *p* = 0.02), with moderate heterogeneity among studies (I^2^ = 72%, *p* = 0.02) (Figure 4, Panel Β).

##### Plasma ET-1 Levels between PEXG Patients vs. Healthy Controls

Among studies included in the meta-analysis, two studies identified examining plasma ET-1 levels between PEXG patients and healthy controls, with a total of 89 subjects (36 PEXG patients vs. 53 healthy controls). No significant difference in plasma ET-1 levels was observed between the PEXG patients and healthy controls [SMD: 0.31 (95% CI: −0.68–1.31, *p* = 0.54)] (Figure 4, Panel C).

#### 3.3.2. Aqueous Humor ET-1 Levels and Glaucoma

##### Aqueous Humor ET-1 Levels between Glaucoma Patients vs. Healthy Controls

**Figure 5:**
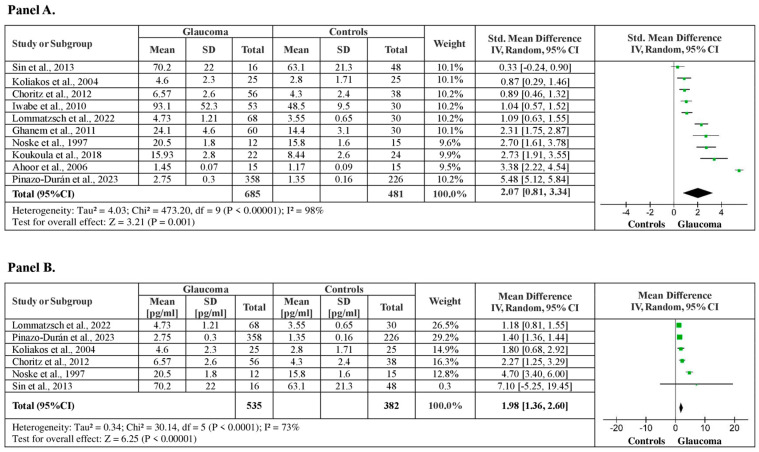
Aqueous humor ET-1 levels between patients with glaucoma and healthy controls. (**A**) Forest plot displaying the meta-analysis of aqueous humor ET-1 levels between glaucomatous patients and control subjects, their estimated pooled standardized mean difference (SMD), confidence interval (CI), and study heterogeneity; (**B**) Forest plot displaying the sensitivity analysis of aqueous humor ET-1 levels between patients with glaucoma and control subjects, their estimated pooled mean difference (MD), confidence interval (CI), and study heterogeneity [17,21,23,25,28,30,33,36,37,38].

From the studies included in the meta-analysis, nine of them examining aqueous humor ET-1 levels between glaucoma patients and healthy controls, with a total of 1166 subjects (685 glaucoma patients vs. 481 healthy controls). The meta-analysis showed significantly higher aqueous humor ET-1 levels for glaucoma patients, by a pooled SMD of 2.07 (95% CI: 0.81–3.34, *p* < 0.001), with a substantial (I^2^ = 87%) heterogeneity among studies (Figure 5, Panel A). The sensitivity analysis confirmed the results showing higher aqueous humor ET-1 levels for glaucoma patients by a pooled mean difference of 1.98 pg/mL ET-1 (95% CI: 1.36–2.60, *p* < 0.001), after funnel plot evaluation and 5 outlier studies removals, with moderate heterogeneity among studies (I^2^ = 73%, *p* < 0.001) (Figure 5, Panel B).

##### Aqueous Humor ET-1 Levels between POAG Patients vs. Healthy Controls

**Figure 6:**
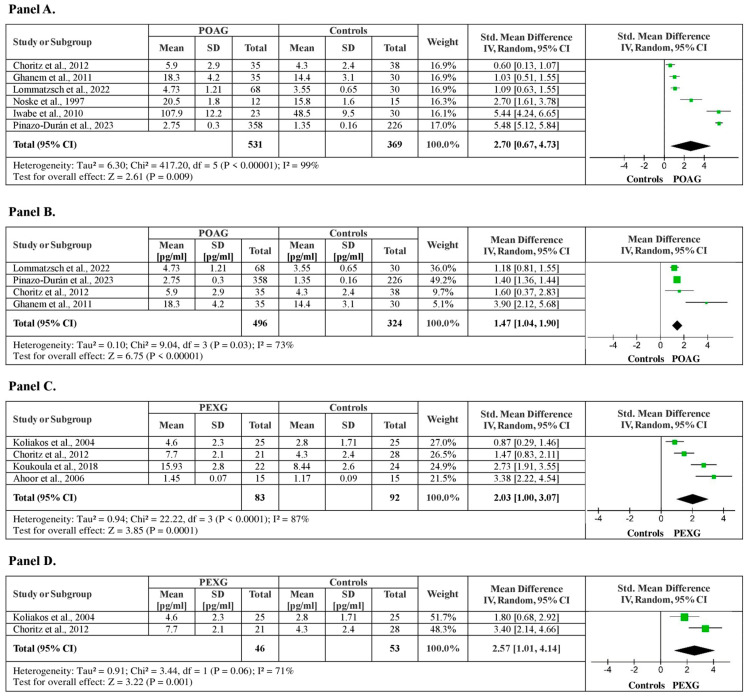
Aqueous humor ET-1 levels between patients with POAG, PEXG, and healthy controls. (**A**) Forest plot displaying the meta-analysis of aqueous humor ET-1 levels between POAG patients and control subjects, their estimated pooled standardized mean difference (SMD), confidence interval (CI), and study heterogeneity; (**B**) Forest plot displaying the sensitivity analysis of aqueous humor ET-1 levels between patients with POAG and control subjects, their estimated pooled mean difference (MD), confidence interval (CI), and study heterogeneity; (**C**) Forest plot displaying the meta-analysis of aqueous humor ET-1 levels between PEXG patients and control subjects, their estimated pooled standardized mean difference (SMD), confidence interval (CI), and study heterogeneity; (**D**) Forest plot displaying the sensitivity analysis of aqueous humor ET-1 levels between patients with PEXG and control subjects, their estimated pooled mean difference (MD), confidence interval (CI), and study heterogeneity [17,21,23,25,28,30,33,36,37].

Five studies were identified investigating aqueous humor ET-1 levels between POAG patients and healthy controls with a total of 900 subjects (531 POAG patients vs. 369 healthy controls). The meta-analysis showed significantly higher aqueous ET-1 levels in POAG patients, by a pooled SMD of 2.70 (95% CI: 0.67–4.73, *p* < 0.001), with a substantial heterogeneity among studies (I^2^ = 99%, *p* < 0.0001) (Figure 6, Panel A). The sensitivity analysis confirmed the results showing higher aqueous humor ET-1 levels for POAG patients by a pooled mean difference of 1.47 pg/mL ET-1 (95% CI: 1.04–1.90, *p* = 0.03), after funnel plot evaluation and two outlier studies removals, with moderate heterogeneity among studies (I^2^ = 73%, *p* = 0 < 0.01) (Figure 6, Panel B).

##### Aqueous Humor ET-1 Levels between PEXG Patients vs. Healthy Controls

Among the studies reviewed, four studies were examining aqueous humor ET-1 levels between PEXG patients and healthy controls, with a total of 175 subjects (83 PEXG patients vs. 92 healthy controls). The analysis showed significantly higher plasma ET-1 levels, by a pooled SMD of 2.03 (95% CI: 1.00–3.07, *p* < 0.001), and high (I^2^ = 87%, *p* < 0.0001) heterogeneity among studies was detected (Figure 6, Panel C). After a sensitivity analysis, a pooled MD of 2.57 pg/mL ET-1 (95% CI: 1.01–4.14, *p* = 0.001) confirmed the results showing higher aqueous humor ET-1 levels in PEXG patients compared to healthy controls, with moderate heterogeneity (I^2^ = 71%, *p* = 0.06) among studies (Figure 6, Panel D). 

## 4. Discussion

This systematic review and meta-analysis have provided a comprehensive evaluation of the role of ET-1 levels in both plasma and aqueous humor among patients with various types of glaucoma compared to healthy controls. Elevated ET-1 has been characterized as a potential causal risk factor for glaucoma development and progression, as several studies in the last two decades have investigated the role of ET-1 levels both in plasma and aqueous humor [30,34,38]. However, the absence of concrete data for the exact relationship of ET-1 levels in each type of glaucoma prompted us to elucidate the exact role it serves among different groups since the last systematic review and meta-analysis by Li S. et al., 2016 included only six studies [40]. 

The significantly elevated levels of ET-1 in the plasma of glaucoma patients observed in our analysis suggest a systemic alteration in vascular regulation among these patients. This aligns with the hypothesis that vascular dysregulation, characterized by an imbalance between vasoconstrictors and vasodilators, may play a crucial role in the development and progression of glaucoma [41,42]. The substantial heterogeneity observed across studies could be attributed to variations in study populations, methodologies, and definitions of glaucoma, which highlights the complexity of this disease and the multifactorial nature of its vascular components [4,43,44]. Interestingly, our findings also revealed significantly higher levels of ET-1 in the aqueous humor of glaucoma patients. This suggests a localized effect of ET-1 within the ocular environment, which may contribute to the impaired ocular blood flow and increased IOP observed in glaucoma patients [45,46,47,48]. The role of ET-1 in regulating ocular blood flow and vessel tone, particularly in the choroid and optic nerve head, provides a plausible mechanism by which elevated ET-1 levels may exacerbate glaucomatous damage [33,49,50,51]. 

Understanding the complexities of trabecular meshwork and its pivotal role in regulating IOP in various glaucoma subtypes is crucial for developing targeted therapies [52]. Several factors such as changes in extracellular matrix metabolism and antioxidant defense system are known to impair aqueous humor outflow [53]. Interestingly, the intravitreal delivery of ET-1 into rat eyes reduced Electroretinography response indicating impaired retinal function and the subsequent decreased blood flow to the retina [54]. Another study supported that mitochondrial dysfunction is a significant contributor to ET-1-induced damage of retinal ganglion cells in the context of POAG, since ET-1 downregulated mitochondrial electron transport chain components, underlining the key mechanism behind the neurodegenerative changes observed in POAG [55]. Moreover, excessive ET-1 in the aqueous humor can lead to abnormal accumulation of extracellular matrix in trabecular meshwork cultured human cells that restricts aqueous humor outflow, thereby increasing IOP [56]. Such tissue-engineered cultures can replicate the morphology and gene expression patterns of specific ocular regions, such as the trabecular meshwork and Schlemm’s canal, providing physiologically relevant models for studying the disease and testing treatments [57]. Recent treatment studies have shed light on these complex interactions and mechanisms within the trabecular meshwork. Particularly, oral administration of macitentan, a dual endothelin receptor antagonist, effectively attenuated the vasoconstriction caused by intravitreal ET-1 and reduced ET-1 induced retinal ganglion cell loss in rats treated with macitentan. Furthermore, intravitreal injection of Magnesium Acetyltaurate (MgAT) provided significant neuroprotective effects against ET-1 induced damage in the retina by suppressing the neuroinflammatory reaction and offering a way to protect against glaucoma-related retinal damage and preserve vision by addressing both vascular dysfunction and oxidative stress [58,59]. Finally, there is a clear need to extend the development of targeted therapies beyond animal models to include human clinical trials, ensuring that treatments addressing ET-1’s role in glaucoma can be effectively translated into therapeutic options that mitigate disease progression.

The analysis across different types of glaucoma revealed that elevated ET-1 levels were not uniform across all forms. Notably, significant elevations were observed in POAG, NTG, and ACG patients, but not in PEXG plasma ET-1 levels compared to healthy controls. This may indicate that the pathophysiological mechanisms involving ET-1 may vary among different types of glaucoma, suggesting the need for type-specific therapeutic approaches. Moreover, the heterogeneity observed in our analyses, particularly in the aqueous humor ET-1 levels, underscores the complexity of glaucoma as a group of diseases and the intricate role of vascular factors in its pathogenesis [43]. The findings of this meta-analysis can be better understood within its limitations, since the considerable heterogeneity among studies indicates differences in studies’ population and the research environment of each work. Future studies should aim to elucidate the mechanisms by which ET-1 contributes to the vascular dysregulation in glaucoma and explore potential therapeutic interventions targeting ET-1 signaling pathways.

## 5. Conclusions

This systematic review and meta-analysis demonstrate that elevated levels of ET-1 in both plasma and aqueous humor are significantly associated with glaucoma, supporting the hypothesis that vascular dysregulation plays a crucial role in its pathogenesis. The findings indicate that ET-1’s impact varies across different types of glaucoma, suggesting the necessity for type-specific research and treatment strategies. Ultimately, understanding the role of ET-1 in glaucoma could lead to novel approaches to treatment and improve outcomes for patients with this challenging ocular disease.

## Figures and Tables

**Table 1:** Characteristics of the included studies.

Study	Country	Population Characteristics	ET-1 Samples Type	Glaucoma Group			Control Group			Keypoints
				*n* =	Mean Age (years)	Male Sex (%)	*n* =	Mean Age (years)	Male Sex (%)	
Sugiyama et al., 1995 [39]	Japan	10 POAG, 52 NTG, and 10 control subjects.	Plasma	62	66.2 yrs.	NA	10	64.8 yrs.	NA	The patients with NTG in the initial stage of visual field loss showed higher plasma ET-1 levels than those in the middle stage respectively. Intravenous administration of ET-1: reduced IOP, reduced blood flow in the optic nerve head, and decreased blood flow in the choroid.
Kaiser et al., 1995 [27]	Switzerland	21 POAG, 10 NTG, and 20 control subjects.	Plasma	31	61.9 yrs.	50.1%	20	60 yrs.	85%	ET-1 plasma levels tended to be higher in NTG patients than in HTG patients and controls. ET-1 plasma levels increased when healthy people change their position from supine to upright.
Cellini et al., 1997 [18]	Italy	15 NTG, and 10 control subjects.	Plasma	15	64.7 yrs.	53.3%	15	65.8 yrs.	60%	ET-1 levels in patients with NTG was significantly higher than in the control group.
Holló et al., 1998 [24]	Hungary	20 POAG, and 44 control subjects.	Plasma	20	61.5 yrs.	55%	44	65 yrs.	38.6%	Plasma ET-1 levels showed no difference among the POAG, Capsular glaucoma and control subjects.
Nicolela et al., 2003 [35]	Canada	41 POAG, and 24 control subjects.	Plasma	41	59.5 yrs.	39%	24	46.9 yrs.	21.2%	Patients with glaucoma, in contrast to the control subjects, had an abnormal increase in plasma levels of ET-1 when they underwent provocative cooling test, despite similar basal plasma ET-1 levels between the two groups.
Kunimatsu et al., 2006 [31]	Japan	18 POAG, 30 NTG, and 19 control subjects.	Plasma	48	47.6 yrs.	63.1%	19	49.9 yrs.	52.6%	In the Japanese population, the plasma ET-1 level showed no difference among NTG patients, POAG patients, and control subjects < 60 years of age.
Ghanem et al., 2011 [23]	Egypt	35 POAG, 25 CCAG, and 30 control subjects.	Plasma, aqueous humor	60	60.6 yrs.	48.3%	30	61.3 yrs.	47%	Aqueous humor ET-1 levels were significantly elevated in CCAG and POAG compared to control subjects. There was no significant difference in plasma levels of ET-1 between the groups studied. NO levels were correlated with ET-1 in the aqueous humor of patients with glaucoma.
Galassi et al., 2011 [22]	Italy	44 NTG, and 40 control subjects.	Plasma	44	64.4 yrs.	54.5%	40	62.7 yrs.	55%	Plasma levels of ET-1 were higher in NTG patients compared with control subjects. In NTG patients, resistivity index of the ophthalmic artery was positively related to ET-1 levels (t = 2.704, *p* = 0.01).
Cellini et al., 2012 [19]	Italy	40 POAG, and 40 control subjects.	Plasma	40	54.5 yrs.	55%	40	52.9 yrs.	50%	POAG patients, compared to healthy controls, showed an increase of ET-1 plasma levels. Increased ET-1 levels in POAG patients were related to vascular dysfunction (r = 0.942; *p* = 0.001) and vascular dysfunction was related to sub-clinical intraocular inflammation (r = 0.968; *p* = 0.001).
Lee et al., 2012 [32]	Korea	45 NTG, and 35 control subjects.	Plasma	35	52.2 yrs.	51.4%	45	53.8 yrs.	48.8%	The plasma ET-1 levels were significantly increased in the NTG group compared with the control group.
Choritz et al., 2012 [21]	Germany	35 POAG, 21 PEXG, and 38 control subjects.	Plasma, aqueous humor	56	67.5 yrs.	46.4%	38	69.7 yrs.	52.6%	ET-1 concentration in aqueous humor were significantly increased in POAG and PEXG compared with controls. No difference was detected for plasma ET-1 concentrations.
Chen et al., 2013 [20]	China	31 POAG, 18 NTG, 16 CCAG, and 37 control subjects.	Plasma	65	52.2 yrs.	75.4%	37	51.9 yrs.	51.3%	Mean ET-1 levels were significantly higher in all 3 of the glaucoma groups (POAG, NTG, CCAG) than in the control group, there was no significant difference in ET-1 level among the 3 glaucoma groups.
López-Riquelme et al., 2015 [34]	Spain	48 POAG, 15 NTG, and 75 control subjects.	Plasma	63	48.8 yrs.	41.2%	75	44 yrs.	22.7%	Plasma levels of ET-1 were significantly higher in the POAG group in comparison with NTG group and controls. NTG patients showed higher ET-1 levels than the healthy controls, but the differences were not statistically significant.
Ahoor et al., 2016 [17]	Iran	15 PEXG, and 15 control subjects.	Plasma, aqueous humor	15	68 yrs.	53.3%	15	66 yrs.	53.3%	Both aqueous and serum levels of ET-1 in the PEXG group were significantly higher than that measured in the control group.
Ji and Jia, 2019 [26]	China	295 POAG, and 200 control subjects.	Plasma	295	58.8 yrs.	55.9%	200	58.7 yrs.	56.5%	POAG patients reported significantly higher plasma levels of ET-1 in comparison with control subjects.
Konieczka et al., 2020 [29]	Poland	34 POAG, 35 NTG, and 35 control subjects.	Plasma	69	66.6 yrs.	32%	34	53.7 yrs.	25%	NTG had significantly higher mean ET-1 plasma levels than the healthy controls. Τhe ET-1 plasma levels increased significantly with age.
Lommatzsch et al., 2022 [33]	Germany	68 POAG, and 30 control subjects.	Plasma, aqueous humor	68	64.5 yrs.	39.7%	30	66.7 yrs.	33.3%	Peripheral ET-1 levels were significantly elevated in patients with glaucoma than in control subjects. ET-1 levels showed the subordinate role of intraocular pressure as a predictive factor for impaired retinal blood flow compared with plasma ET-1 level in glaucoma.
Noske et al., 1997 [36]	Germany	12 POAG, and 15 control subjects.	Aqueous humor	12	NA	NA	15	NA	NA	ET-1 was significantly higher in patients with POAG than in control subjects.
Koliakos et al., 2004 [28]	Greece	25 PEXG, and 25 control subjects.	Aqueous humor	25	70.8 yrs.	40%	25	70.9 yrs.	32%	Mean ET-1 concentration in the PEXG aqueous samples was significantly higher than that measured in the age matched control samples.
Iwabe et al., 2010 [25]	Mexico	53 POAG, and 30 control subjects.	Aqueous humor	53	NA	NA	30	NA	NA	Increased levels of aqueous humor ET-1 levels were reported in POAG patients when compared with control subjects. No statistical difference was observed in aqueous humor ET-1 levels in NTG patients when compared with control subjects.
Sin et al., 2013 [38]	USA	16 NTG, and 48 control subjects.	Aqueous humor	16	66 yrs.	0%	48	65 yrs.	0%	ET-1 levels were significantly higher in eyes with NTG than in control subjects.
Koukoula et al., 2018 [30]	Greece	22 XFG, and 24 control subjects.	Aqueous humor	22	76.2 yrs.	40.9%	24	74.5 yrs.	37.5%	ET-1 aqueous levels in eyes with XFG were significantly higher than those of age-matched controls. ET-1 levels in eyes with XFG exhibit a significant correlation with hemodynamic parameters that indicate reduced perfusion.
Pinazo-Durán et al., 2023 [37]	Spain	358 POAG, and 226 control subjects.	Aqueous humor	226	70.1 yrs.	30%	226	70.6 yrs.	48%	ET1 and NO levels were significantly higher in the aqueous humor from the POAG than that in controls affecting altered modulation of ocular blood flow.

n: number; NA: not available; ET-1: Endothelin-1; POAG: primary open-angle glaucoma; NTG: normal tension glaucoma; IOP: intraocular pressure; HTG: hypertensive glaucoma; CCAG: chronic closed-angle glaucoma; PEXG: pseudoexfoliative glaucoma; XFG: exfoliative glaucoma; yrs.: years.

## Data Availability

All data generated or analyzed during this study are included in this article. Further enquiries can be directed to the corresponding author.

## Supplementary Materials

The following supporting information can be downloaded at: https://www.mdpi.com/article/10.3390/medicina60071117/s1, Table S1: Quality assessment results with the Newcastle-Ottawa Quality Assessment Scale (NOS) tool.
