# Supplementary material for: Endothelin-1 Plasma and Aqueous Humor Levels in Different Types of Glaucoma: A Systematic Review and Meta-Analysis
Source: Medicina (Kaunas). 2024 Jul 10;60(7):1117. doi: 10.3390/medicina60071117 (PMC11278711; doi:10.3390/medicina60071117)
Supplement: Supplementary file 1 [file medicina-60-01117-s001.zip › medicina-3069720-supplementary.pdf]

Supplementary Table 1. Quality assessment results with the Newcastle-Ottawa Quality Assessment Scale (NOS) tool

Study	*Design*	*Selection*	*Comparability*	*Exposure/ Outcome*	*Score*	*Quality*
Sugiyama et al., 1995	case-control	***	*	***	7/9	high
Kaiser et al., 1995	case-control	***	*	**	6/9	moderate
Cellini et al., 1997	case-control	**	*	*	4/9	moderate
Holló et al., 1998	case-control	***	**	***	8/9	high
Nicolela et al., 2003	case-control	***	**	***	8/9	high
Kunimatsu et al., 2006	case-control	****	**	**	8/9	high
Ghanem et al., 2011	cross-sectional	****	**	***	9/10	high
Galassi et al., 2011	case-control	***	**	**	7/9	high
Cellini et al., 2012	case-control	***	*	***	7/9	high
Lee et al., 2012	case-control	****	*	***	8/9	high
Choritz et al., 2012	cross-sectional	****	**	***	9/10	high
Chen et al., 2013	cross-sectional	****	*	***	8/10	high
López-Riquelme et al., 2015	cross-sectional	***	*	***	7/10	high
Ahoor et al., 2016	cross-sectional	***	*	**	6/10	moderate
Ji and Jia, 2019	case-control	***	*	***	7/9	high
Konieczka et al., 2020	case-control	***	**	***	8/9	high
Lommatzsch et al., 2022	case-control	****	**	***	9/9	high
Noske et al., 1997	case-control	**	*	**	5/9	moderate
Koliakos et al., 2004	cross-sectional	****	*	***	8/10	high
Iwabe et al., 2010	case-control	***	*	***	7/9	high
Sin et al., 2013	case-control	***	*	***	7/9	high
Koukoula et al., 2018	cross-sectional	****	*	***	8/10	high
Pinazo-Durán et al., 2023	case-control	****	**	***	10/10	high

Case-Control
Selection (max 4 stars), Comparability (max 2 stars), Exposure (max 3 stars), Score (max score 9)
Cross Sectional
Selection (max 5 stars, Comparability (max 2 stars), Outcome (max 3 stars), Score (max score 10)
Quality
low: 0-3, moderate: 4-6 and high: 7-9 (or 7-10)
